# Genome Sequence of *Bacillus anthracis* Strain Tangail-1 from Bangladesh

**DOI:** 10.1128/genomeA.00748-16

**Published:** 2016-07-28

**Authors:** Farzana Islam Rume, Markus Antwerpen, Peter Braun, Paritosh Kumar Biswas, Mahmuda Yasmin, Gregor Grass, Chowdhury Rafiqul Ahsan, Matthias Hanczaruk

**Affiliations:** a Department of Microbiology, University of Dhaka, Dhaka, Bangladesh; b Bundeswehr Institute of Microbiology, Munich, Germany; c Department of Microbiology and Veterinary Public Health, Chittagong Veterinary and Animal Sciences University, Chittagong, Bangladesh

## Abstract

Soil was collected in July 2013 at a site where a cow infected with anthrax had been the month before. Selective culturing yielded *Bacillus anthracis* strain Tangail-1. Here, we report the draft genome sequence of this *Bacillus anthracis* isolate that belongs to the canonical A.Br.001/002 clade.

## GENOME ANNOUNCEMENT

In Bangladesh, the zoonotic disease anthrax caused by *Bacillus anthracis* is enzootic in various districts of the country (1). For example, from 2008 to 2009 there were a total of 886 registered animal cases and between 18 August and 2 October 2010 alone, 607 human cases were reported (2). In May 2013 an outbreak among livestock occurred in Sadar (Dhaka division), a sub-district of Tangail located in central Bangladesh as published in ProMED-mail (archive 20130517.1720541), where multiple animal cases are typically reported each year. In September 2013, a site from a cowshed housing several healthy cows was sampled and soil from 5 cm depth withdrawn for cultivation. Isolation of live *B. anthracis* was accomplished using the ground anthrax bacillus refined isolation (GABRI) method published previously (3).

Strain Tangail-1 harbored both *B. anthracis* virulence plasmids pXO1 and pXO2 as confirmed by real-time PCR assays (4, 5). Genotyping based on canonical single-nucleotide polymorphism (canSNP) (6) grouped strain Tangail-1 into the A.Br.001/002 branch, which has previously been isolated in Bangladesh (2) and other South Asian countries including China (6, 7), and Central Europe (8, 9). Notably, this canSNP-group of *B. anthracis* seems to be predominant in Bangladesh (1, 2).

Whole-genome shotgun (WGS) sequencing of *B. anthracis* Tangail-1 was performed by Ion Torrent sequencing technology (Ion Torrent Systems Inc., USA). For the WGS library, 1,705,145 reads with a total of 460 Mbases were generated. Bowtie-2 (10) was used for mapping to Ames Ancestor chromosome, plasmid pXO1 and pXO2 (NC_007530.2, NC_007322.2, AE017335.3), respectively. The G+C content was calculated using an in-house Python script.

The total length of the genome shotgun sequence of *B. anthracis* Tangail-1 was 5,227,292 bp with a 105-fold coverage for the chromosome (197-fold for pXO1 and 130-fold for pXO2), and the mean G+C content was 35%. For initial annotation, assembled contigs were submitted to the RAST annotation pipeline (11, 12). The *B. anthracis* Tangail-1 draft genome encodes 5,720 putative coding sequences (CDS). Annotation identified 11 copies of genes for 16S rRNA, 5S rRNA, and 23S rRNA within the genome; 95 tRNA loci were identified. The *B. anthracis* Tangail-1 genome represents a reference genome of a *B. anthracis* strain of the A.Br.001/002-clade from Bangladesh for further country-wide genotype analysis.

### Nucleotide sequence accession numbers.

This whole-genome shotgun project has been deposited at DDBJ/EMBL/GenBank under the accession no. CP015777 (pXO1), CP015778 (pXO2), and CP015779 (chromosome). The versions described in this paper are the first versions.
